# Massachusetts Board of Registration in Dentistry—Resolutions of Respect

**Published:** 1903-01

**Authors:** 


### MASSACHUSETTS BOARD OF REGISTRATION IN DENTISTRY—RESOLUTIONS OF RESPECT.

At a meeting of the Massachusetts Board of Registration in Dentistry the following resolutions were passed:

Whereas, In the sudden death of Dr. J. Searle Hurlbut, which occurred November 9, 1902, the Massachusetts Board of Registration in Dentistry mourns the loss of one of the original members, who for nine years of its existence served with exceptional distinction, four years of which he was president.

*Resolved,* That as an examiner he displayed remarkable wisdom, fairness, and judgment, and showed wonderful tact in his dealing with men.

*Resolved,* That in his contact with his associates on the board, his kindness of nature and generosity of heart will always be remembered with the warmest affection.

*Resolved,* That we extend our sincere and heartfelt sympathy to his bereaved widow in this sad affliction.

*Resolved,* That these resolutions be entered on the records and a copy be sent to the widow of the deceased and to the several dental journals for publication.

(Signed) JOHN F. DOWSLEY.
GEO. E. MITCHELL.
THOS. J. BARRETT.
DWIGHT M. CLAPP.
GEO. A. MAXFIELD.

November 24, 1902.

